# Survey context and question wording affects self reported annoyance due to road traffic noise: a comparison between two cross-sectional studies

**DOI:** 10.1186/1476-069X-11-14

**Published:** 2012-03-11

**Authors:** Theo Bodin, Jonas Björk, Evy Öhrström, Jonas Ardö, Maria Albin

**Affiliations:** 1 Department of Occupational and Environmental Medicine, Skane University Hospital, SE-221 85 Lund, Sweden; 2 Sweden and Lund University, SE-221 85 Lund, Sweden; 3 Competence Center for Clinical Research, Skane University Hospital, SE-221 85 Lund, Sweden; 4 Department of Occupational and Environmental medicine, The Sahlgrenska Academy at the University of Gothenburg, SE-405 30 Gothenburg, Sweden; 5 Department of Physical Geography and Ecosystems Analysis, Lund University, SE-221 85 Lund, Sweden

**Keywords:** Road traffic noise, Railway Noise, Public Health Survey, Survey Methodology, Annoyance

## Abstract

**Background:**

Surveys are a common way to measure annoyance due to road traffic noise, but the method has some draw-backs. Survey context, question wording and answer alternatives could affect participation and answers and could have implications when comparing studies and/or performing pooled analyses. The aim of this study was to investigate the difference in annoyance reporting due to road traffic noise in two types of surveys of which one was introduced broadly and the other with the clearly stated aim of investigating noise and health.

**Methods:**

Data was collected from two surveys carried out in the municipality of Malmö, southern Sweden in 2007 and 2008 (n = 2612 and n = 3810). The first survey stated an aim of investigating residential environmental exposure, especially noise and health. The second survey was a broad public health survey stating a broader aim. The two surveys had comparable questions regarding noise annoyance, although one used a 5-point scale and the other a 4-point scale. We used geographic information systems (GIS) to assess the average road and railway noise (L_Aeq,24h_) at the participants' residential address. Logistic regression was used to calculate odds ratios for annoyance in relation to noise exposure.

**Results:**

Annoyance at least once a week due to road traffic noise was significantly more prevalent in the survey investigating environment and health compared to the public health survey at levels > 45 dB(A), but not at lower exposure levels. However no differences in annoyance were found when comparing the extreme alternatives "never" and "every day". In the study investigating environment and health, "Noise sensitive" persons were more likely to readily respond to the survey and were more annoyed by road traffic noise compared to the other participants in that survey.

**Conclusions:**

The differences in annoyance reporting between the two surveys were mainly due to different scales, suggesting that extreme alternatives are to prefer before dichotomization when comparing results between the two. Although some findings suggested that noise-sensitive individuals were more likely to respond to the survey investigating noise and health, we could not find convincing evidence that contextual differences affected either answers or participation.

## Background

Annoyance caused by traffic noise is a common problem in urban population world wide [1]. Noise derived from aircraft, train and road traffic has increased over the years and is predicted to increase by 23-27% in Sweden during the period 2001 to 2020 [2]. Traffic noise is associated to many adverse effects on life quality and health, including annoyance, disturbance of sleep. In recent years, several studies have also found associations to cardiovascular diseases, such as increased risk of hypertension and myocardial infarction [3,4]. Studies within this field have been many and used a variety of methods to assess both exposure and effects. In some studies, contradictory results have emerged, depending on method [5-7]. Some authors argue that subjective assessment of sleep quality is more relevant than e.g. measuring movements, EEG, blood pressure or other advanced techniques. On the other hand some researchers have chosen to question the validity of self- reporting [8].

Self reporting has many potential sources of bias and several factors influence the respondent's interest in participating. The leverage-saliency theory of survey participation describes how different factors such as cash and other rewards, community involvement, identity of the sender, and personal interest in the survey's topic affect response rates [9]. Personal interest in the survey topic has been shown to increase the response rate with as much as 14% [10]. Recent studies within social science have shown that the differences between responders and non-responders are negligible regarding many commonly asked questions, however demographic items such as income and education, have been skewed [11,12].

There are also factors that do not only affect participation, but also the answers provided by the participants. In social science, it has been shown that participants over- and under-report on certain topics depending on the sender, e.g. under-reporting of revenues in surveys by the tax office [13]. This has, to our knowledge, not been investigated within the field of environmental epidemiology. It would also be plausible that annoyance reporting would be biased by the subject's awareness that the survey is concerned with environmental noise. This has been investigated by others but has not been found to affect answers [14]. The same study did however show that question wording affected reporting. A question where symptoms were explicitly attributed to a noise source "Has aircraft noise made you feel nervous or irritable?" was correlated to noise exposure, whereas "During the past two weeks, have you been nervous or irritable?" was not.

Various noise annoyance scales have been used over the years but since 1997 there is a ISO/TS standard question to assess noise from traffic [15]. In one recent meta-analysis it was found that the use of a numerical 11-point scale was associated with higher reported noise annoyance compared to a verbal 4-point or 5-point scale. However no significant differences were found between 4, 5 or 10 point verbal scales [16].

Annoyance due to noise is influenced by several individual attitudes. A large review described five different fears that affected degree of annoyance: fear of danger from the noise source, noise prevention beliefs, general noise sensitivity, beliefs about the importance of the noise source, and annoyance with non-noise impacts of the noise source. Other demographic characteristics, such as age, sex and education could not be found to affect annoyance [17].

The aim of the present study was to investigate the difference in annoyance reporting due to road traffic noise between two types of surveys, one general public health survey with a wide scope and the other with a clearly stated aim of investigating noise and health. Our hypothesis was that context (type of survey) and personal attributes would affect the responses.

## Methods

This paper has been prepared in accordance to the 1997 ICBEN guidelines for reporting core information from community noise reaction surveys [18].

### Study population and selection

The study population was taken from two different surveys conducted in the Scania region, Sweden. Both surveys were conducted in accordance with Swedish law of ethics. The first survey (Env&Health07), "Undersökning om boendemiljö och hälsa" ("Survey regarding residential environment and health") was sent to 5600 individuals aged 18-79 residing in Malmö on April 12, 2007 (N = 207 781). Answers were collected during the period June-August 2007. The selection was made through a random sampling of 800 individuals from six different strata based on road traffic and railway noise exposure levels using a simplified version of the Nordic prediction model [19,20]. The six strata were based on three levels of road traffic noise (< 40dB(A); 40-60dB(A) and > 60dB(A)) with or without measurable levels of railway noise exposure. One extra stratum consisting of an additional 800 individuals was added based on those living nearby construction sites related to a major railway tunnel project (Citytunneln). These persons were however not included in this study. The response rate was 54.3%.

The second survey (PHSurvey08), was an extensive public health survey in the Scania region in southern Sweden including 134 questions [18]. All persons 18-80 years old, living in this region on 30 June 2004, constituted the study population (N = 855,599). The population was stratified by gender and geographical area, resulting in 2 * 62 = 124 different strata. The stratified samples were randomly selected from the population registry with approximately an equal number of individuals from each stratum. The total sample consisted of 56 000 individuals and those with home address in the municipality of Malmö were included in this study (n = 8000). The data collection begun in the end of August and was finished in November. The final response rate was 49.3% in the sub-selection of Malmö.

### Description of the two different surveys

Env&Health07 was, with some modifications, adapted from Öhrström et al. [21] The title "Residential environment and health" was printed on the title page and the attached introductory letter informed the respondent that the questionnaire contained a "great deal of questions regarding your dwelling and the near environment as well as your own perception of the environment, especially sound and noise".

The 51 questions were designed to assess A) Housing and living conditions (e.g. type of dwelling, surrounding environment, satisfaction with area); B) Annoyance due to environmental exposure (Noise, smell, fumes, vibrations and noise from neighbours); C) Annoyance due to road traffic and railway noise (including effects on everyday life such as radio and TV listening, conversations, sleep and rest); D) Health conditions (hearing impairment, asthma, hypertension, mental health); E) Sleep and rest; F) Basic facts, work and education.

PHSurvey08 was a broad public health survey stating the aim of "/.../getting a current picture of the health of Scanias population and their living- and environmental conditions." The questionnaire was said to "/.../contain questions about your health status and key determinants of health." The 134 questions were divided in sections regarding self-reported illness, general health status, use of prescribed and recreational drugs, sleep and mental well-being, dental health, life-style habits such as smoking, alcohol consumption, physical exercise and diet, sexual health, social relations, violence and threats, occupation and work environment, residential environment, life quality, health care usage, and finally; basic facts such as educational level, place of birth and economy.

### Comparable questions

The two studies included some identical questions and some questions that were similar to each other. Table 1 shows the complete questions and answers regarding noise annoyance used for comparison in this study. All other questions with answers in both English translation and Swedish are found in the appendix.

**Table 1:** The compared questions regarding noise annoyance.

Env&Health07	PHSurvey08
How often are you disturbed or troubled by noise from train indoors in your home?	During the past 3 months, have you felt disturbed by any of the following in or near your housing?
- every day *	a. Sound from neighbors
- Several times a week *	b. Road traffic noise
- Once or twice a week *	c. Train noise
- Once or twice a month or less often	d. Aircraft noise
- Never	e. Car exhausts
	f. Wood-buring smoke
	g. Odor from industries
*Hur ofta störs eller besväras Du av buller från tågtrafik inomhus i Din bostad?*	
- Varje dag*	- Yes, at least once a day*
- Flera gånger per vecka*	- Yes, at least once per week*
- Någon gång per vecka*	- Yes, less often
- *Någon gång i månaden eller mera sällan*	- No, never
- *Aldrig*	
How often are you disturbed or troubled by noise from road traffic indoors in your home?	*Har du de senaste 3 månaderna känt Dig besvärad av något av följande i eller i närheten av Din bostad?*
- every day*	*a. Ljud från grannar*
- Several times a week*	*b. Vägtrafikbuller*
- Once or twice a week*	*c. Tågbuller*
- Once or twice a month or less often	*d. Flygbuller*
- Never	*e. Bilavgaser*
	*f. Vedeldningsrök*
	*g. Lukt från industrier*
*Hur ofta störs eller besväras Du av buller från vägtrafik inomhus i Din bostad?*	
- Varje dag*	- Ja, minst en gång per dag*
- Flera gånger per vecka*	- Ja, minst en gång per vecka*
- Någon gång per vecka*	- *Ja, mer sällan*
- *Någon gång i månaden eller mera sällan*	- *Nej, aldrig*
- *Aldrig*	
* defines dichotomization " Annoyed by road traffic/railway noise 1/w "	* defines dichotomization " Annoyed by road traffic/railway noise 1/w "

Complete questions and answer alternatives in English and *Swedish (italic) *For complete Q&A of questions used, see Additional file 1: Table S1

### Exposure assessment

No measurements of noise levels were conducted. Instead, we used a geographic information system (GIS) to assess the outdoor noise exposure from traffic. Current residential addresses for the participants in both surveys and road traffic data were geocoded. Original road traffic data from the whole region included 21,397 road segments (17,339 administrated by the Swedish Road Administration, and 4,058 by local municipalities). The number of vehicles was available for 82% of the road segments. Speed limits were available for > 95% of the segments. For road segments without traffic data, mean values were assigned to each segment on the basis of existing data for the included road types [22]. Using the road traffic data, we used a simplified version of the Nordic prediction method for road traffic noise [see the reports by Lyse Nielsen [19] and Jonasson et al [23] for a complete description] to estimate noise exposure at the residential locations of the participants. In short, the Nordic prediction method first calculates the unattenuated noise level 10 meters from the road centre using the number of light and heavy vehicles and the speed limit of each road segment. Corrections were then calculated for (i) the distance between the source (the road) and receptor, for which the noise levels decrease by 3 dB(A) with a doubling of the distance, (ii) attenuation due to ground surface type and noise barriers [the attenuation of noise depends on surface type with less attenuation for hard surfaces (asphalt, water, concrete) and more attenuation for soft surfaces (vegetation, grass, etc)], and (iii) additional corrections for special cases (including very steep topography, reflection from buildings, etc).

In this study, we had to simplify the Nordic prediction method by using corrections for distance and surface type only. We were not able to correct for noise barriers and the additional special cases already mentioned, as no such data was available. We assumed flat ground in all cases and soft surfaces between the residence and the road for the participants living in the countryside, while a hard surface was assumed for the participants living in more densely populated areas. We had no data indicating the floor of the apartment building on which the residences were located, and we therefore estimated the noise level on the ground floor for all of the residences.

We estimated the A-weighted equivalent sound level over a full day (24 hours, L_Aeq,24h_) in dB(A). Estimated noise levels during the day and night were too strongly correlated with the noise level during a full day to be used for separate analyses. Using the number of vehicles (light and heavy) and the speed limit for each road segment, we calculated L_Aeq,24h _for each 25-meter zone up to 500 meters from the centre of the road. As subjects may appear in noise zones for more than one road segment, the maximum values for L_Aeq,24h _across all of the road segments near the residence were extracted for each person and used for further analyses. Hence exposure refers to the most exposed façade of the residence.

Exposure levels were cut off at 68 dB(A) for road traffic which was the highest exposure level in PHSurvey08. 37 individuals in Env&Health07 were found to be exposed to levels above 68 dB(A) and were subsequently excluded from the study.

Railway noise exposure was estimated according to the Nordic Prediction method for railroad Noise [20] using a level of detail comparable to the estimation of road noise, see Liljewalch-Fogelmark, 2006 for details [24].

### Statistical analysis

Standard statistical methods were applied using PASW 18.0.1 for Windows (SPSS Inc, Chicago IL, USA). We used the same procedure for analyzing results from both studies. Logistic regression with dichotomized annoyance as outcome variable (defined by the two survey questions marked with * in Table 1) with average road traffic noise exposure during a full day (L_Aeq,24h_) entered as a continuous 1dB(A)-step or categorical variable in 5 dB(A)-intervals. The highest noise levels, ranging from 60 to 68 dB(A), were merged into one group (+60). Reference category for both the continuous and the categorical exposure variable was all subjects with average road noise exposure below 40 dB(A). Logistic regression models were preformed both unadjusted, and adjusted for road traffic and railway noise as well as age, sex, educational level and country of origin. Effect estimates were presented as odds ratios (ORs) with 95% confidence intervals (CIs). Prevalences for annoyance at least once per week were calculated, in both studies, stratified by noise exposure in 5dB(A) groups. 95% Confidence intervals for the estimated prevalences were calculated according to Wilson [25] and differences in prevalence between the two studies were calculated with 95% confidence intervals according to Newcombe model 10 for unpaired data [26].

## Results

Minor demographic differences were found between the respondents in the two surveys including factors known to influence response rate such as age, sex, education, country of birth as well as global health scoring, smoking, marital status and economic situation. (Table 2). Due to differences in sampling procedure, exposure to railway and road traffic noise showed substantial differences. Especially railway noise was uncommon among respondents in PHSurvey08 compared to those in Env&Health07. Table 2 shows the proportion of respondents exposed to high ≥5BA) and low (< 55dB(A)) levels of noise exposure from both sources.

**Table 2:** Comparison of the two studies regarding socio-demographic factors and noise exposure

	PHSurvey08		Env&Health07	
	**n= **	**median (1q-3q)**	**n= **	**median (1q-3q)**

Age	3810	48 (33-62)	2612	46 (33-61)

BMI	3702	24.8 (22.4-27.7)	2542	24.6 (22.2-27.5)

Health and well-being (7p-scale)	3755	5 (4-6)	2609	5 (4-6)

	n=	Percentage	n=	Percentage

Born abroad	3810	25%	2574	26%

Men	3810	45%	2612	46%

Smokers	3764	21%	2539	25%

Married/co-living	3810	60%	2558	67%

University education	3521	40%	2612	43%

Strained economy	3709	8%	2555	8%

Exposed to railway noise 5	3810	9%	2612	48%

Exposed to road traffic noise 5	3810	15%	2596	33%

We wanted to investigate the response pattern in Env&Health07 to determine whether socio-demographic factors, noise exposure and noise sensitivity influenced responses. All responses were registered at return and time-stamped. Using this variable we could stratify the responses by week of return. Table 3 shows the cumulative response for every second week after the survey was sent out and a break-down by age, sex and noise related factors. A logistic regression model was constructed with response within two weeks (readily reply) vs. later reply as outcome. Both an un-adjusted and adjusted model was calculated. Women responded significantly more readily than men. The same was true for older compared to younger individuals. Respondents who characterised themselves as "noise sensitive" were found to be more likely to readily reply than non-sensitive individuals, OR (95%CI) 1.25 (1.04-1.49) in a fully adjusted model.

**Table 3:** Cumulative percentage and number of respondents by time to respond to questionnaire.

					WEEK					Response	OR for readily reply (95%CI)	
		**1-2**		**3-4**		**5-6**		**7+**		**rate**	**unadjusted**	adjusted*

**Env/Health07**	Men	55%	(661)	78%	(265)	88%	(125)	100%	(141)	50%		

	Women	60%	(851)	81%	(296)	88%	(106)	100%	(167)	59%	1.22 (1.04-1.43)	1.23 (1.04-1.44)

	Age: 19-29	48%	(231)	75%	(132)	88%	(63)	100%	(57)	40%		

	Age: 30-49	53%	(513)	77%	(236)	89%	(111)	100%	(111)	52%	1.23 (0.98-1.53)	1.28 (1.02-1.60)

	Age: 50-65	65%	(451)	82%	(114)	88%	(42)	100%	(86)	64%	1.99 (1.56-2.53)	1.96 (1.53-2.52)

	Age: 66-79	68%	(317)	85%	(79)	88%	(15)	100%	(54)	72%	2.33 (1.78-3.05)	2.27 (1.69-3.04)

	Noise exposure < 55 (road+railway)	60%	(540)	80%	(181)	88%	(69)	100%	(110)	62%		

	Noise exposure 5 (road+railway)	57%	(972)	79%	(380)	88%	(162)	100%	(198)	51%	0.87 (0.73-1.03)	1.38 (0.97-1.97)

	Annoyed by road traffic noise < 1/w	59%	(944)	80%	(338)	88%	(131)	100%	(185)			

	Annoyed by road traffic noise 1w	56%	(523)	78%	(203)	88%	(93)	100%	(112)		0.89 (0.76-1.05)	1.12 (0.93-1.34)

	Annoyed by railway noise < 1/w	59%	(1229)	80%	(433)	88%	(177)	100%	(247)			

	Annoyed by railway noise 1w	54%	(218)	78%	(99)	88%	(40)	100%	(47)		0.82 (0.66-1.02)	0.98 (0.77-1.25)

	Not noise sensitive	57%	(982)	79%	(383)	88%	(155)	100%	(211)			

	Noise sensitive	60%	(503)	80%	(163)	89%	(74)	100%	(92)		1.17 (0.99-1.39)	1.26 (1.05-1.50)

**PHSurvey08**	Noise exposure < 55 (road+railway)	19%	(569)	71%	(1539)	86%	(433)	100%	(426)			

	Noise exposure 5 (road+railway)	19%	(162)	69%	(418)	85%	(134)	100%	(129)		0.97 (0.80-1.19)	1.25 (0.95-1.66)

	Annoyed by road traffic noise < 1/w	19%	(507)	70%	(1321)	85%	(371)	100%	(402)			

	Annoyed by road traffic noise 1w	20%	(211)	73%	(572)	88%	(162)	100%	(124)		0.99 (0.82-1.19)	1.06 (0.87-1.29)

	Annoyed by railway noise < 1/w	20%	(674)	71%	(1769)	86%	(505)	100%	(495)			

	Annoyed by railway noise 1w	19%	(42)	73%	(120)	87%	(30)	100%	(29)		0.92 (0.65-1.32)	1.06 (0.73-1.55)


* Adjusted for railway and road traffic noise, age, sex, educational level and country of birth

Comparison by road traffic and railway noise annoyance and exposure

**Figure 1:**
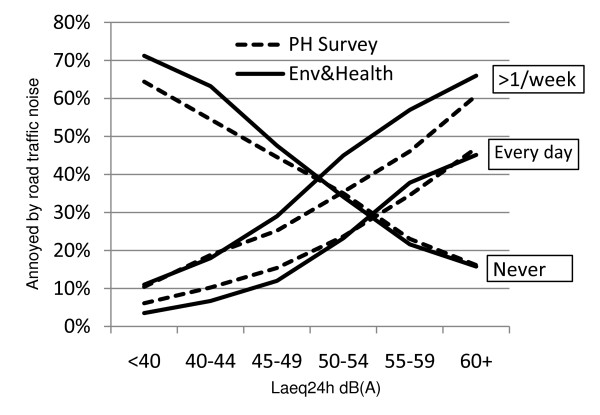
**Proportion of respondents annoyed by road traffic noise more than once per week in relation to noise exposure**. Also extreme alternatives in questionnaire presented. "Every day" (ascending line) and "Never" (descending line).

Annoyance due to road traffic noise was investigated in both studies (Table 1). Table 4 shows the proportion being annoyed at different levels of noise exposure. No apparent difference was found between the two studies regarding the proportion of respondents being annoyed every day or among those never being annoyed. When the answer alternatives were dichotomised into two groups (annoyed at least once a week and annoyed less often) we found that respondents in Env&Health07 reported more frequent annoyance than those in PHSurvey08 at exposure levels exceeding 45dB(A). (Figure 1)

**Table 4:** Annoyance frequency by road traffic noise exposure.

	L_Aeq,24h _dB(A) ROAD						
**PHSurvey08**	**< 40**	**40-44**	**45-49**	**50-54**	**55-59**	**60+**	**Total**

At least once per day	6%	10%	15%	24%	35%	47%	19%

At least once per week	4%	9%	10%	12%	12%	14%	10%

Less often	25%	27%	30%	30%	31%	23%	29%

Never	64%	54%	45%	35%	23%	16%	42%

**Env&Health07**							

Every day	4%	7%	12%	23%	38%	45%	21%

Several times/w	2%	3%	6%	7%	9%	8%	6%

Once or twice per w	5%	8%	11%	14%	11%	13%	10%

Once or twice per month or less	18%	19%	24%	21%	21%	18%	20%

Never	71%	63%	48%	34%	22%	16%	43%

Not sensitive to noise	9%	15%	23%	37%	51%	55%	30%

Sensitive to noise	15%	27%	40%	61%	69%	85%	52%

Comparison between the two studies. For Env&Health 07 annoyance more than once per week is also reported for noise-sensitive and non-sensitive persons

Using the method proposed by Newcomb 1998 for measuring proportion differences between two unpaired samples, differences between Env&Health07 and PHSurvey08 with 95% confidence intervals were 10% (4-16), 11% (2-20) and 5% (-3-15 (n.s)) respectively for the highest exposure stratum.

Railway noise exposure differed markedly between the two populations, so we performed further analysis excluding high exposures to improve comparability. When excluding all respondents who were exposed to railway noise levels exceeding 50 dB(A) in both studies results remained largely unchanged.

In Env&Health07, annoyance due to road traffic noise was higher among persons who described themselves as "quite sensitive" or "very sensitive" to noise compared to non-sensitive individuals ("not so sensitive" and "not sensitive at all"). (Table 4)

## Discussion

### Principal findings

We found a strong positive relation between road traffic noise and annoyance in both studies. Baseline prevalence of annoyance at least once per week was the same in both studies up to L_Aeq,24h _40-44 dB(A). However, at noise levels exceeding 45dB(A), participants in the study explicitly investigating the relation between traffic noise and health (Env&Health07), were more likely to report annoyance more than once per week due to road traffic noise, compared to those participating in the broadly aimed public health survey (PHSurvey08), also when taking differences in railway noise exposure into account. However, no apparent difference was found when comparing the proportion of respondents being annoyed every day or among those never being annoyed.

Noise sensitivity was associated with readily reply (within two weeks) to the Env&Health07 which implicates that there might be a context based participation bias. However, we lacked information concerning noise sensitivity from the other study (PHSurvey08), and also failed to find any additional evidence supporting the assumption that context influenced the answers.

### Strengths and limitations of the study

There were only small differences between the two populations regarding demographic factors such as age, sex and country of birth. Health-related items such as smoking, BMI, and over-all health showed negligible differences. The prevalence of experiencing economical difficulties was comparable, as was educational level, while civil status slightly differed between the two populations. However, the sampling method differed between the two studies which might have some implications for the results. We weighted the studies with regard to the stratifying variables (sex and geographical area in PHSurvey08 and geographical area in Env&Health07), but found no significant changes in the main results in this analysis.

One year passed between the two studies, during which some changes in exposure might have occurred. However, we believe that these changes are of lesser importance. More likely to affect exposure is timing of the year. Others have shown that seasonal differences in annoyance may account for a small proportion of noise annoyance variability with annoyance being higher in the summer than in winter-time [27]. However, the two surveys in this study were sent out to the respondents at roughly the same time of the year. Although some difference in wording, the questions also covered the same seasonal period, hence, making them comparable in this regard.

Unfortunately we could not analyze the population reduction in relation to noise exposure in the PHSurvey08 because we lacked exposure assessments for the non responders. Neither did PHSurvey08 include questions regarding noise sensitivity. This makes it difficult to validate findings regarding participation bias due to noise sensitivity or noise exposure.

Noise annoyance should preferably be measured using the ISO/TS-certified questions [15,28] to facilitate comparisons between results in socio-acoustical surveys. The questions compared in this study have not been validated by others and may have caused additional misclassification on top of the difference in wording between the two surveys.

Our assessment of road traffic noise exposure was based on actual data on traffic intensity for a majority of the road segments. The same model was used in both surveys. Data on vehicles for road segments belonging to the municipality was included. A limitation was that we did not have data on noise barriers (including buildings), window glassing and floor level which is of interest in urban areas. Preliminary results from an ongoing study in Scania's largest urban area (Malmö) show that the simplified Nordic prediction model (see methods section) overestimates the exposure compared to a gold-standard model. The median difference was +1dB(A); Quartiles: -3, 7 dB(A); 2.5-97.5 percentiles: -10, 18 dB(A) (n = 2,966) with a slight trend towards larger over-estimations at higher noise levels [29]. Comparing our road noise estimates to recent "state of the art" noise estimates for Malmö city by Ingemansson [30] reveal some deviations, (R^2 ^= 0.23, RMSE = 17 dB(A), mean absolute difference = 14.7 dB(A), n = 4528), potentially influenced by the fact that Ingemansson estimated noise levels in 5dB(A) classes compared to our continues estimates. The absolute deviation was > 5dB(A) for 3990 points and > 10 dB(A) for 2920 points out of a total of 4528 sample points.

The precision error is of classical type [31]. All above mentioned flaws in the simplified model are most likely to lead to an underestimation of our results and might have implications on lower noise levels. Reassuringly, effects on the categorical analysis where our main findings lay should be marginal, whereas the continuous analysis might suffer more from the precision error. On the other hand, we observed a clear correlation between modelled exposure and self-reported annoyance from road traffic noise, indicating a reasonable ranking of current exposure across study subjects.

We only had data on the current residential address, which means that the exposure assessment does not necessarily reflect long-term exposure. However, most subjects (60% in both surveys) had lived at least five years at the current address. Average noise exposure < 40 dB(A) was used as reference category. The prevalence of annoyance, as shown in table 4, increased between < 40 and 40-44 dB(A) which supports that exposures below current guidelines could be of some importance. This was also shown for the whole region of Scania in a study by us a few years ago [32]. Others have suggested that exposure assessments below LAeq,24h 45dB(A) could be less reliable [33,34]. Our exposure methods also showed higher overestimation for points with low noise level estimates according to the "gold standard" method.

### Results in relation to previous studies

Our findings regarding readily reply and annoyance among noise sensitive individuals in Env&Health07 indicate that participation bias may be one of the reasons for the differences in annoyance reporting between the studies. Noise sensitive individuals would, because of their sensitiveness, have a greater interest in participation compared to a non-sensitive person, analogous to findings by others studying reasons for survey participation [10]. Readily response have been investigated by others as a predictor for participation bias and it has been found that late responders are more similar to non-responders [35]. This is also supported by the findings in this study, that women respond more readily and have a higher response rate than men. The same was found when considering response time and rate in relation to age. Noise sensitive individuals report, as we know, more annoyance compared to non-sensitive (Table 4 and [17]). Hence, it is plausible that two studies, whereof one with the explicit aim of investigating traffic noise and health and the other with a broader aim, would produce different results regarding noise annoyance. Although annoyance frequency differed when dichotomizing at more or less than once a week, comparison of the extreme alternatives "Every day" and "Never" showed no difference between the two studies.

Hence, we believe that different number of alternatives did produce different results. This was proposed in data supplied by Rohrmann to the ICBEN [28], and also shown in a study by Yano in 1997 with results consistent with ours suggesting that the extreme alternatives showed better alignment between scales than dichotomized variables [36]. As illustrated in Figure 1, no difference was found between the two studies at low noise exposure levels. These findings suggest that that the number of alternatives could matter more at high exposure levels.

Other possible explanations to the difference could be recall bias or fatigue. In the public health survey with a large number of questions, one could fear that respondents might respond with less scrutiny closer to the end, producing falsely low annoyance prevalence, although it has been reported that annoyance question position in a survey does not affect the answers [28].

### Implications for further studies

Inter-study comparability has been a crucial goal within the field of noise and health. The guidelines developed, and standardized survey questions have made this easier over the last two decades but difficulties when comparing have not seized to exist. The topic of this study is rarely studied within the field of environmental epidemiology, although surveys are a very common way to estimate impact of various exposures. This study has several limitations but raises an important call for further research and methodological development in survey-based noise effects studies and meta-analysis. We would like to see further studies with the ability to compare responses to the ISO/TS 15666 questions, thus isolating the possible importance of context as proposed in this article without the wording or number of alternatives affecting the results. Based on this study's main finding, that dichotomized variables are not comparable when the number of alternatives differs, we would recommend using the extreme categories when comparing two surveys with different sets of questions.

## Conclusions

The wording and number of alternatives on the annoyance reporting was found to produce different results when comparing the two studies. This has been suggested earlier by others, but to our knowledge never investigated properly. Although our hypothesis included that noise-affected and noise-sensitive individuals would participate and/or answer differently to a survey investigating noise and health compared to survey with a wide scope, we could not, due to the limitations in data, find convincing evidence that these contextual differences affected either answers or participation.

## Abbreviations

BMI: Body mass index (weight in kg divided by height in meters squared); GIS: Geographical information system; dB(A): A-weighted sound level in decibel; L_Aeq,24h _: Day-night average sound level; OR: Odds ratio; 95% CI: 95% confidence interval.

## Competing interests

The authors declare that they have no competing interests.

## Authors' contributions

All authors of this paper have read and approved the final version submitted. They have also directly participated either in the planning, execution, or analysis of this study. TB did the statistical analysis and drafted the paper. MA developed the study design together with JB and EÖ (Env&Health07). JA did the GIS modeling and wrote parts of the paper's method section. All authors have revised drafts and contributed to the discussion.

## Supplementary Material

Additional file 1 **Table S1**. List of comparable questions in the two studies. Complete questions and answer alternatives in English and *Swedish (italic)*. Click here for file
