# Supplementary material for: Survey context and question wording affects self reported annoyance due to road traffic noise: a comparison between two cross-sectional studies
Source: Environ Health. 2012 Mar 11;11:14. doi: 10.1186/1476-069X-11-14 (PMC3315434; doi:10.1186/1476-069X-11-14)
Supplement: Additional file 1 — Table S1. List of comparable questions in the two studies. Complete questions and answer alternatives in English and Swedish (italic). [file 1476-069X-11-14-S1.DOC]

| **Table 1:** List of comparable questions in the two studies. Complete questions and answer alternatives in English and *Swedish (italic)* | |
| --- | --- |
| **Env&Health07** | **PHSurvey08** |
| In which year were you born? *Vilket år är du född?* | In which year were you born? *Vilket år är du född?* |
| Are you a woman or a man?  *Är Du kvinna eller man?* | Are you a woman or a man?  *Är Du kvinna eller man?* |
| Were you born in Sweden?  *Är du född i Sverige?* | Were you born in Sweden?  *Är du född i Sverige?* |
| How tall are you?  *Hur lång är du?* | How tall are you?  *Hur lång är du?* |
| How much do you weigh?  *Hur mycket väger du?* | How much do you weigh?  *Hur mycket väger du?* |
| Your marital status?  Married / cohabiting  Unmarried / not cohabiting  *Ditt civilstånd?*  *Gift/sammanboende*  *Ogift/ej sammanboende* | What is your marital status?   1. Married / partner 2. Unmarried 3. Divorced 4. Widow / widower   *Vilket är ditt civilstånd?*   1. *Gift/sambo* 2. *Ogift* 3. *Frånskild* 4. *Änka/änkling* |
| How do you feel right now, physically and mentally, in the aspect of your health and well-being   - 7 point scale   *Hur känner Du Dig just nu, fysiskt och psykiskt, om Du ser till Din hälsa och Ditt välbefinnande?*   - *Kryssa i lämplig ruta mellan 1 - 7 (1 = mycket dåligt, 7 = mycket bra)* | How do you feel right now, physically and mentally, in the aspect of your health and well-being   - 7 point scale   *Hur känner Du Dig just nu, fysiskt och psykiskt, om Du ser till Din hälsa och Ditt välbefinnande?*   - *Kryssa i lämplig ruta mellan 1 - 7 (1 = mycket dåligt, 7 = mycket bra)* |
| Do you smoke?   - Yes, every day   How many cigarettes/cigars/pipes/day?   - - - 1-7/day     - 8-15/day     - 16-25/day     - More than 25/day - Yes, but not every day - No, I stopped smoking   - Less than 1 year ago   - 1 to 5 years ago   - More than 5 years ago - No, I have never been a smoker   *Röker Du?*   - *Ja, dagligen*    - *Hur många cigaretter/*   *cigarrer /pipstopp/dag?*   - - - *1-7/dag*     - *8-15/dag*     - *16-25/dag*     - *mer än 25/dag* - *Ja, men inte dagligen* - *Nej, jag har slutat för*    - *mindre än 1 år sedan*   - *1 till 5 år sedan*   - *mer än 5 år sedan* - *Nej, jag har aldrig rökt* | 41. Do you smoke?  - Yes, daily  - Yes, but not every day 🡪 42  - No 🡪 42  If YES DAILY: How much do you smoke on average?   - Cigarettes daily: XX - Cigarillos daily: XX - Cigars daily: XX - Grams of pipe tobacco a week: XX   42 a) Have you ever smoked daily for at least 6 months?   - No. - Yes   b) If YES: For how long ago did you smoke a day?   - Less than 6 months ago - Between 6 and 12 months ago - More than one year ago, indicate the year: XXXX   *41. Röker Du?*   - *Ja, dagligen* - *Ja, men inte alla dagar 🡪 42* - *Nej 🡪 42*   *Om JA DAGLIGEN: Hur mycket röker Du i genomsnitt?*   - *cigaretter dagligen*: XX - *cigarriller dagligen*: XX - *cigarrer dagligen*: XX - *gram piptobak i veckan*: XX   *42. a) Har Du tidigare rökt dagligen under minst 6 månader?*   - *Nej* - *Ja*   *b) Om JA: För hur länge sedan slutade Du röka dagligen?*   - *Mindre än 6 månader sedan* - *Mellan 6 och 12 månader sedan* - *Mer än 1 år sedan, ange vilket år: XXXX* |
| How long has your education been?  A. Primary school, High school or similar  1-8 years (eg, elementary school)   - 9 years (eg primary school) - 10-11 years (eg, primary + 2-year high school) - 12-13 years (eg, primary school + 3- or 4-year high school)   B. University - or college   - Less than 3 years - 3 years or more - No university or college   *Hur lång utbildning har Du?*  *A. Grundskola, gymnasium eller liknande*   - *1-8 år (t ex folkskola)* - *9 år ( t ex grundskola)* - *10-11 år (t ex grundskola + 2-årigt gymnasium)* - *12-13 år (t ex grundskola + 3- eller 4-årigt gymnasium)*   *B. Universitets- eller högskoleutbildning*   - *Kortare än 3 år* - *3 år eller mer* - *Ingen universitets- eller högskoleutbildning* | What is the highest education you have?  If you are studying right now tick the planned degree. Just mark one alternative!   - Elementary school or elementary school - “Real skola” or girls' school - 2-year upper secondary school or vocational school - 3-4-year upper secondary - University or college, less than 3 years (less than 150ECTS credits) - University or college, 3 years or more (180 ECTS credits or more) - Other Education, which one? Write in the box:   *Vilken är den högsta utbildningen Du har?*  *Om Du studerar kryssa i den utbildning Du går. Sätt bara ett kryss!*   - *Folkskola eller grundskola* - *Realskola eller flickskola* - *2-årigt gymnasium eller yrkesskola* - *3-4-årigt gymnasium* - *Universitet eller högskola, mindre än 3 år (mindre än 100 p)* - *Universitet eller högskola, 3 år eller längre (120 p eller mer)* - *Annan utbildning, vilken? Skriv i rutan:* |
| How often has it happened during the past 12 months that you have had difficulties in paying your bills (rent, electricity, telephone, rates, mortgage, insurance, etc.)?   - Every month - About half of the months in a year - Occasionally - Never   *Hur ofta har det hänt att Du under de senaste 12 månaderna haft svårigheter att klara Dina*  *räkningar (hyra, el, telefon, räntor, amorteringar, försäkringar etc)?*   - *Varje månad* - *Ungefär hälften av årets månader* - *Någon enstaka gång* - *Aldrig* | How often has it happened during the past 12 months that you have had difficulties in paying your bills (rent, electricity, telephone, rates, mortgage, insurance, etc.)?   - Every month - About half of the months in a year - Occasionally - Never   *Hur ofta har det hänt att Du under de senaste 12 månaderna haft svårigheter att klara Dina*  *räkningar (hyra, el, telefon, räntor, amorteringar, försäkringar etc)?*   - *Varje månad* - *Ungefär hälften av årets månader* - *Någon enstaka gång*   *Aldrig* |
| How often are you disturbed or troubled by noise from train indoors in your home?   - every day * - Several times a week * - Once or twice a week * - Once or twice a month or less often - Never   *Hur ofta störs eller besväras Du av buller från tågtrafik inomhus i Din bostad?*   - *Varje dag** - *Flera gånger per vecka** - *Någon gång per vecka** - *Någon gång i månaden eller mera sällan* - *Aldrig*   How often are you disturbed or troubled by noise from road traffic indoors in your home?   - every day* - Several times a week* - Once or twice a week* - Once or twice a month or less often - Never   *Hur ofta störs eller besväras Du av buller från vägtrafik inomhus i Din bostad?*   - *Varje dag** - *Flera gånger per vecka** - *Någon gång per vecka** - *Någon gång i månaden eller mera sällan* - *Aldrig*   * defines dichotomization “Annoyed by road traffic / railway noise ≥1/w “ | During the past 3 months, have you felt disturbed by any of the following in or near your  housing?  a. Sound from neighbors  b. Road traffic noise  c. Train noise  d. Aircraft noise  e. Car exhausts  f. Wood-buring smoke  g. Odor from industries   - Yes, at least once a day* - Yes, at least once per week* - Yes, less often - No, never   *Har du de senaste 3 månaderna känt Dig besvärad av något av följande i eller i närheten av Din*  *bostad?*  *a. Ljud från grannar*  *b. Vägtrafikbuller*  *c. Tågbuller*  *d. Flygbuller*  *e. Bilavgaser*  *f. Vedeldningsrök*  *g. Lukt från industrier*   - *Ja, minst en gång per dag** - *Ja, minst en gång per vecka** - *Ja, mer sällan* - *Nej, aldrig*   * defines dichotomization “Annoyed by road traffic / railway noise ≥1/w “ |
